# Nutriomes and nutrient arrays - the key to personalised nutrition for DNA damage prevention and cancer growth control

**DOI:** 10.1186/2041-9414-1-11

**Published:** 2010-08-12

**Authors:** Michael F Fenech

**Affiliations:** 1 CSIRO Food and Nutritional Sciences, PO Box 10041 Adelaide BC, SA, 5000. Australia

## Abstract

DNA damage at the base-sequence, epigenome and chromosome level is a fundamental cause of developmental and degenerative diseases. Multiple micronutrients and their interactions with the inherited and/or acquired genome determine DNA damage and genomic instability rates. The challenge is to identify for each individual the combination of micronutrients and their doses (i.e. the nutriome) that optimises genome stability and DNA repair. In this paper I describe and propose the use of high-throughput nutrient array systems with high content analysis diagnostics of DNA damage, cell death and cell growth for defining, on an individual basis, the optimal nutriome for DNA damage prevention and cancer growth control.

## Background and Current Status

DNA damage at the base sequence, epigenome and chromosome level is the most fundamental cause of developmental and degenerative diseases (including accelerated ageing) and is predictive prospectively of these conditions [1-3]. Hundreds of genes are involved in maintenance of genome integrity and there is great variation amongst individuals with respect to common polymorphisms that impact on the activity of these enzymes [4-8]. The proteins encoded by those genes required for DNA replication, DNA repair or detoxification of potential genotoxins depend on essential cofactors that are obtained from the diet for optimal function [1,9] (Table 1; [9-24]). Dietary profile differs between individuals to varying extents depending on their acquired or inherited dietary preferences and food availability; furthermore uptake of micronutrients from the digestive system and transport into cells of the body also varies depending on genetics and altered expression of transporters that occurs with age [25,26]. Nutritional factors are not only required for genome maintenance *in vivo *but also *in vitro *which varies greatly depending on the culture medium used [27,28]. Maintenance of genome integrity *in vitro *is critical particularly in long term culture of cells (e.g. stem cells) which may be taken out of the body for expansion and then returned to the original donor or other recipients for medical therapy reasons because DNA damage accumulated *in vitro *may result in oncogenic events in stem cells [29,30]. Currently dietary reference values (e.g. recommended daily intakes, upper safety limits) and culture medium recipes and conditions do not take into consideration impact on genome integrity and yet harm to the DNA sequence and/or the epigenome is the most fundamental and critical pathology underlying cellular and organism health and disease.

**Table 1:** Examples of the role and the effect of deficiency of specific micronutrients on genomic stability [9-24]

Micronutrient/s	Role in genomic stability	Consequence of deficiency
Vitamin C, Vitamin E, antioxidant polyphenols (e.g. caffeic acid)	Prevention of oxidation to DNA and lipid oxidation.	Increased base-line level of DNA strand breaks, chromosome breaks and oxidative DNA lesions and lipid peroxide adducts on DNA.

Folate and Vitamins B2, B6 and B12	Maintenance methylation of DNA; synthesis of dTMP from dUMP and efficient recycling of folate.	Uracil misincorporation in DNA, increased chromosome breaks and DNA hypomethylation.

Niacin	Required as substrate for poly(ADP-ribose) polymerase (PARP) which is involved in cleavage and rejoining of DNA and telomere length maintenance.	Increased level of unrepaired nicks in DNA, increased chromosome breaks and rearrangements, and sensitivity to mutagens.

Zinc	Required as a co-factor for Cu/Zn superoxide dismutase, endonuclease IV, function of p53, Fapy glycosylase and in Zn finger proteins such as PARP.	Increased DNA oxidation, DNA breaks and elevated chromosome damage rate.

Iron	Required as component of ribonucleotide reductase and mitochondrial cytochromes.	Reduced DNA repair capacity and increased propensity for oxidative damage to mitochondrial DNA.

Magnesium	Required as co-factor for a variety of DNA polymerases, in nucleotide excision repair, base excision repair and mismatch repair. Essential for microtubule polymerization and chromosome segregation.	Reduced fidelity of DNA replication. Reduced DNA repair capacity. Chromosome segregation errors.

Manganese	Required as a component of mitochondrial Mn superoxide dismutase.	Increase susceptibility to superoxide damage to mitochondrial DNA and reduced resistance to radiation-induced damage to nuclear DNA.

Calcium	Required as cofactor for regulation of the mitotic process and chromosome segregation.	Mitotic dysfunction and chromosome segregation errors.

Selenium	Selenoproteins involved in methionine metabolism and antioxidant metabolism (e.g. selenomethionine, glutathione peroxidase I).	Increase in DNA strand breaks, DNA oxidation and telomere shortening.

## Needs and Knowledge Gaps

A critical issue in tissue culture is the evident lack of physiological conditions both in terms of composition of culture medium as well as oxygen tension both of which have profound impacts on the rate of growth of cells and their level of chromosomal instability. For example recipes of culture media can vary enormously between each other with respect to minerals and vitamins and often the concentration is supra-physiological relative to human serum or deficient depending on the micronutrient. RPMI 1640 culture medium, one of the most commonly used for culturing human cells, is supra-physiological for folate, methionine and riboflavin and deficient for iron, copper, zinc, calcium, magnesium and sulphur relative to human serum (Table 2). While some of the deficiencies in culture medium may be addressed by addition of foetal bovine serum this is only added at 5-10% which would still render culture medium deficient if the micronutrient is absent or deficient in the recipe. It is evident that current culture media are not physiological relative to human plasma and therefore data obtained from *in vitro *experiments need to be treated with caution if attempts are made to extrapolate to *in vivo *predictions. The latter can only become feasible once physiological culture media are developed that are equivalent in composition to human plasma and other body fluids (e.g. cerebro-spinal fluid, interstitial fluid) and if the oxygen tension used is similar to that experienced by tissues in the body. Physiological oxygen tension is at least 2-4 times lower than that of atmospheric oxygen typically used in cell culture incubators; it was shown that cells grown under physiological oxygen conditions experience less oxidative stress and paradoxically grow more slowly compared to cells in atmospheric oxygen incubators [31,32]. Faster growth does not necessarily result in better genome stability because the former could be due to permissiveness of cell cycle checkpoints causing a reduction in cell cycle time and/or reduced apoptosis of cells with DNA damage.

**Table 2:** Comparison of concentration of some micronutrients between a single sample of human serum and normal complete RPMI1640 culture medium (data not previously published).

Micronutrient	Concentration unit	Human serum	RPMI 1640 culture medium
Folate	μmol/L	0.028	2.3

Methionine	μmol/L	30	100

Riboflavin	μmol/L	0.05	0.53

Iron	mg/L	0.84	0.19

Copper	mg/L	1.4	<0.1

Zinc	mg/L	0.94	0.17

Calcium	mg/L	98	26

Magnesium	mg/L	20	11

Sodium	mg/L	3400	3200

Potassium	mg/L	154	200

Phosphorous	mg/L	121	174

Sulphur	mg/L	1110	64

We and others have shown that DNA damage, cell death and cell growth in cultured cells are strongly affected by concentration of essential micronutrients such that both deficiency or excess within the physiological range can profoundly harm the genome and alter cell growth and survival kinetics [19,27,28,33,34]. The use of excessively high concentrations of methyl donors (e.g. folate, methionine, choline vitamin B12) in culture medium theoretically may lead to an adverse DNA methylation pattern that may inappropriately silence important house-keeping genes although strong evidence for this hypothesis is currently lacking [34]. It is evident that, given the wide spectrum of micronutrients required for genome maintenance and repair, the development of physiological culture medium composition is an important pre-requisite to enable the determination of optimal culture conditions for growth of human cells in a genomically stable state and to explore the impact of various micronutrient combinations (i.e. nutriomes) and dosages against different genetic backgrounds. These developments are also critical if we are to use *in vitro *data reliably to predict *in vivo *nutritional effects on an individual basis. In this regard it is important to note that concentrations of micronutrients achievable *in vitro *might not be possible *in vivo *due to excretion and re-distribution within tissues. Furthermore, with respect to body fluids, we only have good knowledge on possible micronutrient concentrations in blood plasma and our knowledge about interstitial fluids surrounding organs (e.g. cerebrospinal fluid) or within tissues is at this stage rudimentary. We need to consider optima both within the physiological and supra-physiological range but only use "physiological dose ranges" achievable *in vivo *for *in vivo *predictions.

With respect to optimising *in vitro *and *in vivo *cellular health it is becoming increasingly recognised that parameters of genome and epigenome damage are exquisitely sensitive to changes in micronutrient concentration even within the "normal" physiological range [19,27,28,34-36]. It is therefore practical, feasible and desirable to start re-examining dietary reference values so that recommended intakes coincide with the attainment of tissue concentrations that are consistent with minimised DNA damage. For a detailed recent review on the status of validation of DNA damage biomarkers for measuring the genomic impact of malnutrition and a proposed roadmap for determining nutrient and nutriome requirements for optimal genome maintenance refer to Fenech 2010 [1].

## Testing Nutriomes in Nutrient Arrays

**Figure 1:**
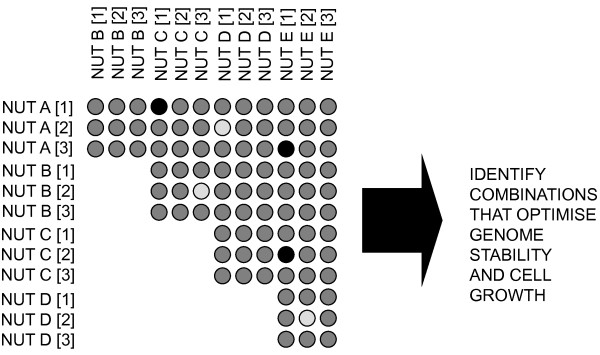
**Nutrient arrays - The Rosetta Stone for unlocking personalised nutrition for genome maintenance**. Theoretical example of a simple Nutrient Array microculture system. NUT = single nutrient or multiple nutrient combination; A-E = different types of nutrients or nutrient combinations; 1-3 = increasing dose levels. The different grey level colouring is simply an indication of the potential variability in cell growth, viability and genome stability that may be observed depending on the combinations used. The challenge is to identify the best combination or combinations for each individual.

The biggest challenge in nutritional genomics is to make the quantum leap from a reductionist single nutrient-single gene interaction approach to studying the interaction of the complete nutrient combination (i.e. the nutriome) with the whole genome on an individual by individual basis. The ultimate goal is effectively to find for each individual the nutriome that best matches their genome so that cellular function and genome and epigenome maintenance is optimised. The "Rosetta Stone" (mechanism or code) to unravel this puzzle lies in developing nutrient arrays in microculture systems such that multiple nutriomes can be simultaneously tested whilst taking into consideration impact of dosage in the assessment (figure 1). The microwell that produces cells than can proliferate adequately and viably whilst maintaining optimal genome and epigenome stability is likely to represent the best nutriome match for that individual's cells. The development of high content automated analyses of DNA damage has already become feasible using quantitative image cytometry [37-40] such that multiple measures can be captured simultaneously in interphase cells including the number of cells and their nuclear DNA content, multiple measures of genome stability such as telomere length and aneuploidy by FISH, oxidised guanine and DNA methylation by immunohistochemistry, chromosome damage and telomere end fusions by micronucleus cytome assays in cytokinesis-blocked binuncleated cells and so on.

**Figure 2:**
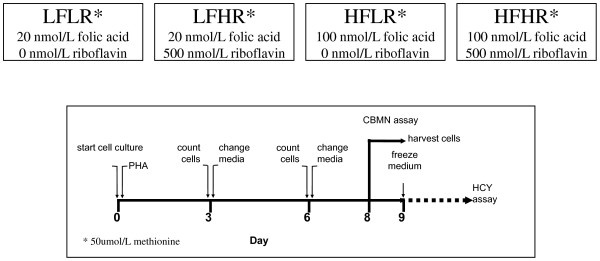
**Experimental Design**. A schematic diagram of a simple nutrient array design that was used to study the interactive effects of folic acid (F) and riboflavin (R) at low (L) and high (H) physiological concentration. In this study by Kimura et al 2004 [28] the folic acid-riboflavin interactive effects in four different combinations (LFLR, LFHR, HFLR, HFHR) on DNA damage were measured using the CBMNcyt assay in lymphocytes that were homozygous for the common or rarer allele of the C677T polymorphism in the methylenetetrahydrofolatereductase (*MTHFR*) gene. Riboflavin is a precursor of the FAD cofactor for MTHFR and folic acid is a precursor for 5,10-methylenetetrahydrofolate the substrate for MTHFR. Cell growth and homocysteine were also measured. **N = 7 C677C, N = 7 T677T**

**Figure 3:**
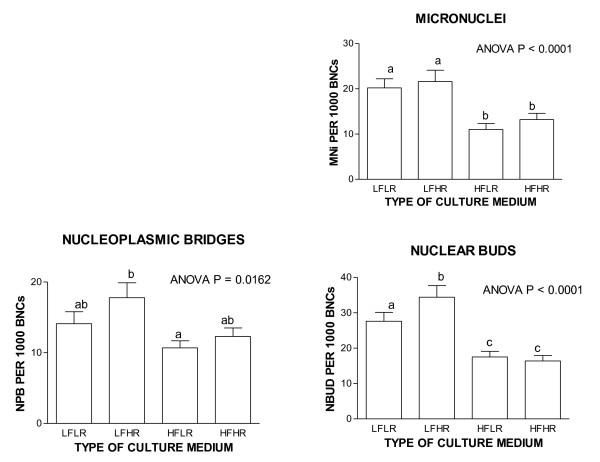
**High riboflavin tends to increase genome instability when folate status is low**. Folate-riboflavin interactive effects on CBMNcyt assay DNA damage biomarkers. It is evident from these results that folate deficiency tends to increase micronuclei, nucleoplasmic bridges and nuclear buds and these effects are further aggravated by high riboflavin in a low folic acid background. For more details refer to Kimura et al 2004 [28]. L, low; H, high; F, folic acid; R, riboflavin.

**Figure 4:**
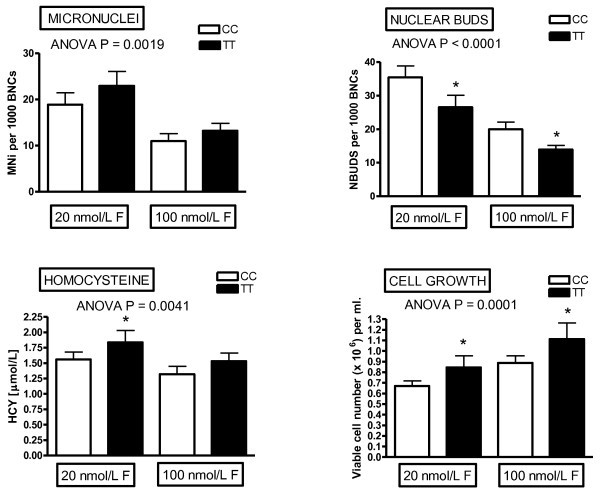
**Effect of MTHFR C677C and T677T genotype on DNA damage, homocysteine and cell growth**. The differential effects of the *MTHFR *C677T genotype on micronuclei, nuclear buds, homocysteine and cell growth. It is evident from the results of this study that homozygous carriers of the T allele tended to have higher levels of micronuclei, homocysteine and cell growth and lower frequencies of nuclear buds relative to homozygous carriers of the common C allele. For more details refer to Kimura et al 2004 [28]. * P < 0.05 relative to corresponding CC

**Figure 5:**
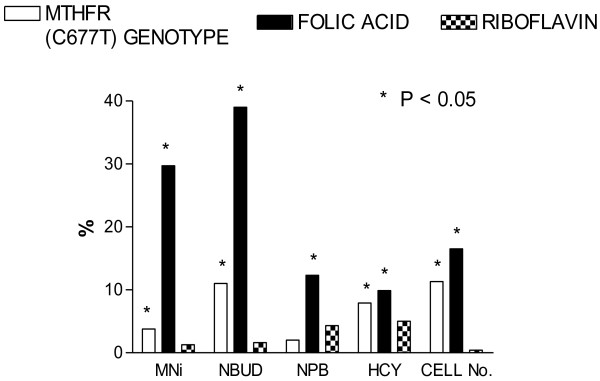
**Percentage of variation of DNA damage biomarkers, homocysteine and cell growth explained by genotype, folic acid or riboflavin**. Percentage variation of micronucleus, nucleoplasmic bridge, nuclear bud frequency, homocysteine concentration and cell growth explained by folic acid or riboflavin concentration or *MTHFR *C677T genotype. It is evident that folic acid is the dominant variable with respect to DNA damage biomarkers, whilst *MTHFR *C677T genotype was almost as important as folic acid in explaining the degree of variation for homocysteine and cell growth. Riboflavin only appeared to have some substantial but small effect on the variation of nucleoplasmic bridges and homocysteine. For more details refer to Kimura et al 2004 [28].

Such a system would also identify the deficiency and safe upper limit range for that individual for multiple micronutrients within a single scan and identify any unexpected combinations that could prove counter-intuitively cytotoxic or genotoxic. The plausibility of such a possibility is supported by our observation that genome instability increased under low folate conditions (20 nM) if riboflavin concentration was increased to replete status [28] (figures 2, 3, 4, 5) possibly because the latter, which is the precursor of the FAD cofactor for MTHFR, increases MTHFR activity which catalyses the irreversible conversion of 5,10methylenetetrahydrofolate to 5-methyltetrahydrofolate making the former folate species less bioavailable for dTMP synthesis from dUMP and thus increasing uracil in DNA. Excessive uracil in DNA causes abasic sites and DNA strand breaks when uracil glycosylases attempt to repair this highly mutagenic lesion [35,41,42]. Therefore it is important to develop a nutrient array system that can efficiently interrogate multiple micronutrient combinations at different dosages. This type of approach has the added advantage that it becomes possible to identify an individual's nutriome for genome health maintenance without needing to know the person's genetic background. Furthermore, such systems could also be used to compare the response of different genotypes under the same nutriome conditions and estimate the percentage of the variance of the biomarkers measured that are explained by different genotype and different nutrients in the nutriomes tested including their interactions (see figures 2, 3, 4, 5 as an example for the effects of the MTHFR C677T genotype relative to folic acid and riboflavin on DNA damage, homocysteine and cell growth biomarkers).

Prototypes of this approach have been designed by our group and others to investigate the following interactive effects on DNA damage, cell death and cell growth:

(i) different ratios of sulphur- and seleno-methionine at constant physiological methionine concentration [33].

(ii) folate concentration with alcohol [43].

(iii) alcohol/acetaldehyde concentration and ADH1 or ALDH2 genotype [44,45].

(iii) folate concentration with BRCA1 or BRCA2 genotype [46,47].

(iv) folate concentration with riboflavin concentration with MTHFR C677T genotype [28].

In these studies the CBMNcyt assay was used to obtain multiple measures of chromosomal instability, cell death and cell division [48,49]. The results of this approach are very promising because not only can they readily define the % variation in genotoxicity, cytotoxicity, metabolite and cell growth biomarkers that is attributable to a specific micronutrient, genotype and interactions between these parameters but also define the shape of the nutrient/DNA damage dose-response curve for genetically defined cell types. The use of the CBMNcyt assay is particularly relevant for this purpose because the relative incidence of DNA damage, cell death events and cytostasis varies as micronutrients and their concentrations within a nutriome are increased or decreased in multiple combinations. The relevant nutriomes within a single metabolic pathway may involve more than just two micronutrients; for example the folate-methionine cycle requires folate in various forms as a substrate and betaine, vitamin B12, vitamin B6 and vitamin B2 as co-factors. Therefore, the nutrient array should also be designed to interrogate combinations of multiple micronutrients simultaneously in a dose-related manner and at different or contrasting dosage levels for each micronutrient relative to the others.

The *in vitro *nutrient array system would also be an ideal mechanism to test whether the predictions of emerging nutrigenomic mathematical models in specific key metabolic pathways [50,51] actually hold true because this system is less likely than *in vivo *human models to be affected by problems relating to compliance to dietary intervention and unexpected life-style and exposure variables such as stress and recreational drug consumption as well as environmental genotoxicants which can impact on the genome damage indices measured. Furthermore it is financially prohibitive to test multiple micronutrient combinations *in vivo*.

## The Future

**Figure 6:**
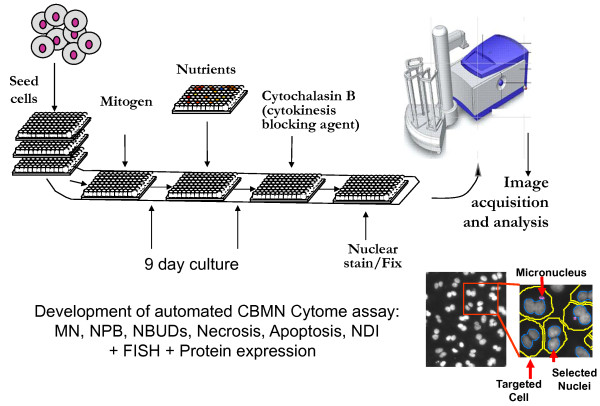
**Minimally invasive high-throughput nutrient array screening for genome-protective agents**. A schematic diagram of the essential components of a high throughput high-content-analysis automated nutrient array system using human peripheral blood lymphocytes.

Realisation of the promise of nutrient array systems is dependent on the following technological developments:

1. Physiological culture systems based on appropriate composition of the culture medium to reflect exactly the actual extracellular fluid composition in diverse tissue environments (e.g. plasma, cerebrospinal fluid) as well as the correct oxygen tension which can modify susceptibility and rate of nuclear and cell division.

2. The issue of nutrient requirements based on cell division kinetics could be significant but remains unexplored. Nutrient array systems will need to be developed for both dividing and non-dividing (confluent) cell cultures including 3 D cultures with mixed populations of dividing and non-dividing cells to test whether nutritional requirements for genome maintenance might vary depending on cell division status.

3. Because of the very large number of possible combinations of micronutrients (vitamins, minerals, phytonutrients, antioxidants and amino acids) a high throughput system is required that will be able to measure cell growth, cell death and DNA damage biomarkers in micro-cultures of cells using high content analyses with live cell-imaging systems that ideally will perform such measurements simultaneously in a manner that is not destructive of cells so that continuous measurements can be performed over several days with minimal cell number requirements (figure 6). The ability to perform continuous measurements over several days or weeks is particularly relevant to nutrition because the effects of micronutrients are chronic rather than acute and their impact could drift as cells adapt to the different nutriome environments that they are exposed to in the nutrient array system.

4. Ideally such systems will be able to interrogate not only the optimal nutritional requirements for growth and genome maintenance of normal cells from an individual but also to verify that such a nutriome does not stimulate growth of cancer cells that the individual might have. Cancer cells are likely to have a markedly different genotype to that of the host's normal cells and could respond differently to the same nutriome environment. For example some cancer cells amplify the high affinity folic acid receptor [52] giving them a distinct advantage over normal cells, when folate is limiting, in accessing folate from the surrounding fluid. The ideal nutriome for an ageing or cancer-prone individual would be the combination that not only sustains the replenishment of normal cells in a genetically integral manner but also inhibits the growth of cancer cells. It is conceivable that both normal cells and cancer cells from an individual could be simultaneously tested within a single nutrient array system.

5. Ultimately, although such systems can be readily implemented for optimising *in vitro *culture conditions of cells, their practical use will be greatly enhanced once they have been validated for predicting the *in vivo *nutritional requirements of an individual. The data from the optimal *in vitro *nutriome, after comparison to plasma concentration, can then be used to estimate deficiencies or excesses of micronutrients in body fluids and appropriate dietary intervention can then be designed to make the necessary adjustments to optimise genome stability. This approach could be used in the emerging integrative and preventive medicine modality based on Genome Health Clinics in which developmental and degenerative diseases are prevented via diagnosis and nutritional prevention of DNA damage [11,53]. Whether such attempts to optimise micronutrient status should be limited to those with above average DNA damage levels will remain an open question until we can find out what is the lowest DNA damage level achievable *in vitro *or *in vivo*.

6. Whether the nutrient array system can be adapted for use directly with an individual's sample of their own body fluids is an important question as this may be a better basis for *in vitro *testing of the efficacy of multiple nutritional adjustments under conditions that reflect exactly the individual's current physiological status. Although this approach seems attractive its feasibility has yet to be explored and could be limited by the difficulty of culturing cells in human serum.

7. There will always be a need to review and revise the DNA damage biomarkers that are most suitable for use in the nutrient array system based on their status of validation. At this point in time the cytokinesis-block micronucleus assay is the best validated with respect to its sensitivity to nutritional status and prospective association with cancer and cardiovascular disease mortality [1,54-57]. Ultimately an automated high content analysis approach that integrates multiple complementary biomarkers of genome damage and instability (e.g. mitochondrial DNA mutations, telomere length, DNA methylation, micronuclei, nucleoplasmic bridges) would be required to achieve a deeper understanding of optimal nutritional requirements for genome maintenance on an individual basis.

8. Peripheral blood lymphocytes are ideal for use in the nutrient array system because they are easy to obtain and culture and have been used extensively to measure DNA damage *in vitro *and *in vivo*. Furthermore, because they travel throughout the body they experience fluctuations in micronutrient concentrations and nutriome profiles that may occur in different tissues and therefore can integrate the genomic impacts of sub-optimal nutrition throughout the body. Whether, the effects in lymphocytes might reflect what would happen in other tissues, such as epithelial tissues, is an important question because it is difficult to culture easily accessible epithelial tissues such as buccal mucosa. Two recent studies suggest that the level of micronuclei in lymphocytes correlates well with micronuclei in buccal cells and with DNA damage in sperm [58,59]. However, despite these promising results more evidence is needed to justify the sole use of lymphocytes in the nutrient array system and ideally a practical epithelial cell culture alternative is also developed in the future.

In conclusion the use of nutrient array systems to interrogate genomic responses to multiple nutrient doses and combinations is in principle feasible and holds great promise to define the nutriome requirements of any cell type to either sustain its growth and reproduction in a genetically stable manner in the case of normal cells and stem cells or to suppress its growth and cause its death in the case of cancer cells.

## Competing interests

The author declares that they have no competing interests.
